# Thalamic GABA Predicts Fine Motor Performance in Manganese-Exposed Smelter Workers

**DOI:** 10.1371/journal.pone.0088220

**Published:** 2014-02-04

**Authors:** Zaiyang Long, Xiang-Rong Li, Jun Xu, Richard A. E. Edden, Wei-Ping Qin, Li-Ling Long, James B. Murdoch, Wei Zheng, Yue-Ming Jiang, Ulrike Dydak

**Affiliations:** 1 School of Health Sciences, Purdue University, West Lafayette, Indiana, United States of America; 2 Department of Radiology and Imaging Sciences, Indiana University School of Medicine, Indianapolis, Indiana, United States of America; 3 Department of Radiology, The First Affiliated Hospital of Guangxi Medical University, Nanning, China; 4 Russell H. Morgan Department of Radiology and Radiological Sciences, The Johns Hopkins University School of Medicine, Baltimore, Maryland, United States of America; 5 FM Kirby Center for Functional Brain Imaging, Kennedy Krieger Institute, Baltimore, Maryland, United States of America; 6 Department of Occupational Poisoning, Guangxi Institute for Occupational Disease Prevention and Treatment, Nanning, China; 7 Toshiba Medical Research Institute USA, Mayfield Village, Ohio, United States of America; 8 Department of Health Toxicology, Guangxi Medical University, Nanning, China; CINVESTAV-IPN, Mexico

## Abstract

Overexposure to manganese (Mn) may lead to parkinsonian symptoms including motor deficits. The main inhibitory neurotransmitter gamma-aminobutyric acid (GABA) is known to play a pivotal role in the regulation and performance of movement. Therefore this study was aimed at testing the hypothesis that an alteration of GABA following Mn exposure may be associated with fine motor performance in occupationally exposed workers and may underlie the mechanism of Mn-induced motor deficits. A cohort of nine Mn-exposed male smelter workers from an Mn-iron alloy factory and 23 gender- and age-matched controls were recruited and underwent neurological exams, magnetic resonance spectroscopy (MRS) measurements, and Purdue pegboard motor testing. Short-echo-time MRS was used to measure N-Acetyl-aspartate (NAA) and myo-inositol (mI). GABA was detected with a MEGA-PRESS J-editing MRS sequence. The mean thalamic GABA level was significantly increased in smelter workers compared to controls (p = 0.009). Multiple linear regression analysis reveals (1) a significant association between the increase in GABA level and the duration of exposure (R^2^ = 0.660, p = 0.039), and (2) significant inverse associations between GABA levels and all Purdue pegboard test scores (for summation of all scores R^2^ = 0.902, p = 0.001) in the smelter workers. In addition, levels of mI were reduced significantly in the thalamus and PCC of smelter workers compared to controls (p = 0.030 and p = 0.009, respectively). In conclusion, our results show clear associations between thalamic GABA levels and fine motor performance. Thus in Mn-exposed subjects, increased thalamic GABA levels may serve as a biomarker for subtle deficits in motor control and may become valuable for early diagnosis of Mn poisoning.

## Introduction

Excessive manganese (Mn) exposure is known to cause Parkinson-like symptoms including cognitive, psychiatric and motor deficits as first described by Couper (1837) and currently referred to as manganism [1], [2]. Exposed individuals may complain about apathy, insomnia, malaise, or diminished libido. Other symptoms may include disorientation, emotional instability, compulsive acts, hallucinations, and slurring and stuttering speech with diminished voice. In severe cases, the patients exhibit extrapyramidal disorders, such as gait imbalance, bradykinesia, or action/postural tremor [3]–[4]. Magnetic resonance imaging (MRI) and spectroscopy (MRS) can be used to evaluate brain Mn accumulation and to measure Mn exposure-induced metabolite changes non-invasively in occupationally exposed workers [5]–[11]. Mn shortens T_1_ relaxation times, and the resulting high MRI signal intensities can be observed in the globus pallidus, striatum, olfactory bulb, and other brain locations [5]–[8]. Correlations between grooved pegboard motor test scores and the Mn-induced intensity of T1-weighted images, a semi-quantitative indicator of Mn accumulation, from frontal grey matter and globus pallidus have been shown in asymptomatic welders [8], [12]. In addition, decreased N-Acetyl-aspartate (NAA, a marker of neuronal function) and myo-inositol (mI, a glial cell marker) were also found in various brain regions [9]–[11].

Gamma-aminobutyric acid (GABA), the main inhibitory neurotransmitter in the central nervous system, is known to play a pivotal role in the regulation and performance of movement [13], [14]. Altered GABA concentrations in the basal ganglia are associated with movement disorders [15], and GABA is known to be involved in the mechanism of motor deficits [16], [17]. For example, increases in striatal GABA levels were first demonstrated in idiopathic Parkinson disease (IPD) in postmortem studies [18], [19]. Recently a high-field in vivo MRS study demonstrated significantly elevated GABA levels in the striatum and pons of IPD patients [20]. Moreover, our group previously found significantly increased GABA levels in a group of active, Mn-exposed smelter workers who had not been clinically diagnosed with any movement deficit [10].

In light of the connection between increased GABA levels and motor deficits, we tested the hypothesis that an alteration of GABA following Mn exposure may underlie Mn-induced motor deficits and that increased brain GABA levels may serve as an additional, non-invasive, metabolite-based marker of effect in early Mn neurotoxicity.

## Methods

### Ethics Statement

This study was approved by the Institutional Review Boards at Purdue University and the Ethical Review Committee at the First Affiliated Hospital of Guangxi Medical University. A written consent form was obtained from each subject prior to the onset of the study.

### Subjects

Nine male Mn-exposed asymptomatic smelter workers, from here on called “smelters”, working in an Mn-Fe alloy factory and 23 age- and gender-matched control subjects with no history of Mn exposure participated in this study. All are residents of Guangxi Province, China. Table 1 summarizes the demographic information. All workers worked 8-hour days for five days a week. The concentrations of airborne Mn were determined in the breathing zones of workers by a stationary air sampler as described in a previous study [7]. Samples were collected in duplicate every other hour for 5 hours in total on the same day. Mn concentrations in air samples were measured using flame atomic absorption spectrometry (Shimadzu Model AA-6800, Japan).

**Table 1:** Exposure information of the Mn-exposed smelters and controls.

	Smelters (n = 9)	Controls (n = 23)
Mean airborne Mn [mg/m^3^]	0.40 ± 0.30	N/A
Mean years of occupational exposure	20.4 ± 6.2	0
Mean age (years)	39.3 ± 7.0	36.9 ± 10.7
Erythrocyte Mn (mg/L)	0.16 ± 0.07*	0.06 ± 0.02
Urine Mn (µg/L)	98.08 ± 126.44*	8.92 ± 3.27

* p<0.05; N/A: lower than test limit of flame atomic absorption spectrometry.

Subjects did not have any history of neurological disorders. Both controls and smelters were asymptomatic, defined as never having sought evaluation or treatment for tremor, Parkinson disease or any motor dysfunction.

All subjects were evaluated by a neurologist specialized in movement disorder at the First Affiliated Hospital of Guangxi Medical University using their standard neurological exam for motor evaluation. Blood and urine samples were collected to measure Mn concentrations in erythrocytes and urine using a model JY-70PII inductively coupled plasma-atomic emission spectrophotometer (ICP-AES, JY70P Type II, Jobin-Yvon Company, France). The detailed procedure has been described in a previous publication [7].

### MRI/MRS and GABA editing

**Figure 1:**
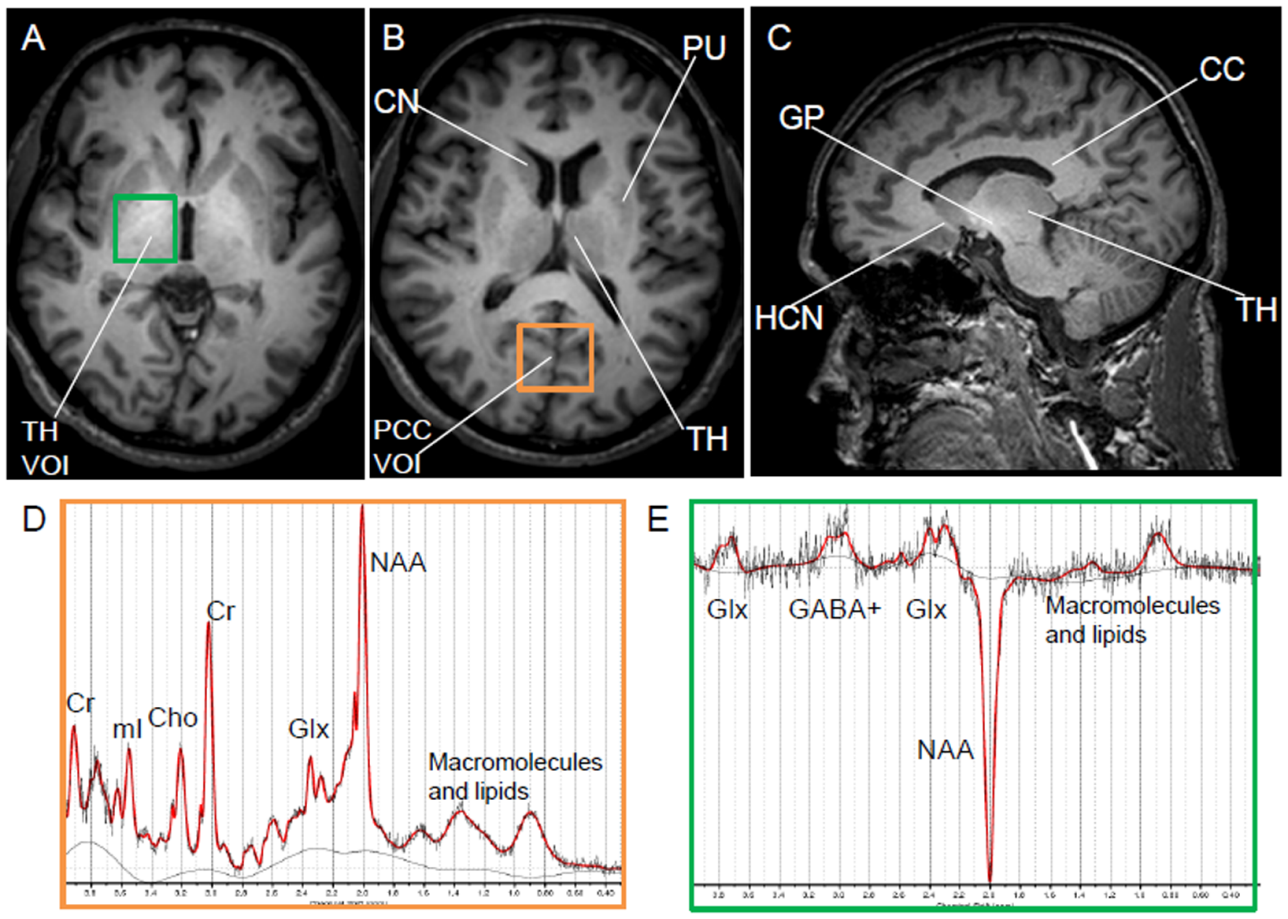
Voxel placement and representative ^1^H short-TE and GABA-edited spectra with LCModel fitting. (A) Volume of interest (VOI) for the thalamus (TH); (B) VOI for the posterior cingulate cortex (PCC); (C) a representative sagittal T1-weighted MRI brain image of a smelter, depicting the hyperintense signal associated with brain manganese deposition, especially in the globus pallidus; (D) a representative short-TE spectrum with LCModel fitting; (E) a representative MEGA-PRESS difference spectrum with LCModel fitting. CC: corpus callosum, Cho: choline, CN: caudate nucleus, Cr: creatine, Glx: combination of glutamate and glutamine, GP: globus pallidus, HCN: head of caudate nucleus, mI: myo-inositol, NAA: N-Acetyl-aspartate, PU: putamen,

MRI scans were performed on a 3T Philips Achieva whole-body clinical scanner (Philips Healthcare, Best, the Netherlands), equipped with an eight-channel head coil. In addition to imaging, short-echo-time (short-TE) ^1^H spectra (PRESS localization; TR/TE = 1500/30 ms; CHESS water suppression) were acquired in each subject from two volumes of interest (VOI): one in the posterior cingulate cortex (PCC) (26.2 ml) and a second centered on thalamus (22.5 ml), but also containing portions of the globus pallidus, putamen and other basal ganglia structures, a location that showed increased GABA levels in our earlier study of another smelter group [10]. An additional GABA-edited proton spectrum was acquired from the thalamus VOI. The positioning of the VOIs and representative spectra are shown in Figure 1. For each MRS scan, a reference spectrum was acquired without water suppression and used later for phase and frequency correction of the corresponding water-suppressed spectrum. Shimming and other preparation phases were performed fully automatically, resulting in line widths of < 15 Hz for the unsuppressed water peak for all spectra.

The MEGA-PRESS J-editing sequence [21], later adapted and optimized by Edden and Barker [22], was used for GABA detection (TR/TE = 2000/68ms). This sequence makes use of the J-coupling between pairs of protons in the GABA molecule to isolate its small signal amidst much larger peaks. 128 averages were acquired with the spectrally selective editing pulse centered at 1.9 ppm and 128 averages with the pulse centered at 7.6 ppm in an interleaved fashion. The resulting difference spectrum contains a GABA peak at 3.0 ppm. This GABA signal measured by MRS cannot distinguish between pre- and postsynaptic GABA concentrations. Another known problem with this version of the MEGA-PRESS technique is that the signal at 3.0 ppm also includes a contribution from co-edited macromolecules (“MM30”). Moreover, *any* GABA measurement by MRS also includes a small contribution from homocarnosine, a dipeptide consisting of GABA and histidine with the same spin behavior as GABA at 3.0 ppm. Hence, the signal that we obtain will be referred to as GABA+.

MRS data processing and quantification were performed with LCModel [23], fitting each spectrum as a weighted linear combination of basis spectra from individual metabolites. For the short-TE data, a basis set of *in vitro* spectra from individual metabolite solutions was used. NAA and mI concentrations were evaluated to focus on neuronal and glial function, respectively. All NAA and mI fitting results had relative standard deviations (%SD) < 10% as reported by LCModel. To cancel out variations in signal due to coil loading or partial volume effects, we present metabolite concentrations as ratios with respect to the total creatine (tCr) concentration. For the MEGA-PRESS spectra, basis sets were generated from density matrix simulations of the sequence using published values for chemical shifts and J-couplings [24], with an exact treatment of metabolite evolution during the two frequency-selective MEGA inversion pulses. Difference basis spectra were obtained by subtracting the simulated metabolite response to selective inversion at 7.6 ppm from that at 1.9 ppm. Because the subtraction was assumed to be perfect, only those metabolites with resonances close to 1.9 ppm were included in the difference basis sets: GABA, glutamate, glutamine, glutathione, and N-Acetyl-aspartate. We relied on the flexible baseline of LCModel to fill in most of the relatively featureless MM resonance at 3.0 ppm, thereby reducing the MM contribution to the overall fitted GABA+ signal (same as “Method 1” in our previous study [10]). As with the short-TE data, we report GABA+/tCr, the ratio of GABA+ to total creatine. tCr levels in the GABA-VOI were obtained from spectra with the MEGA-PRESS editing pulse centered at 7.6 ppm.

### Motor tests

We have previously successfully used the Purdue pegboard test (Lafayette Instrument, Indiana, USA) to study motor function among metal workers [25]. The device has been proven to be sensitive for assessing gross movements of the fingers, hands and arms, as well as fine fingertip dexterity necessary in assembly tasks. In short, each subject was asked to place small pins on a pegboard as quickly as possible with the left hand, right hand, and both hands, or to assemble pins, collars and washers in 1 min. The number of pins inserted or the number of parts assembled was recorded as the test score. A higher score indicates a better command of manual dexterity and better steadiness.

### Statistics

All data are expressed as mean ± standard deviation (S.D.). The data were first tested for a normal distribution and comparison of means between smelter and control groups was performed with a two-tailed Student t-test. Statistical analysis was conducted using PASW Statistics 18.0 software (http://www-01.ibm.com/software/analytics/spss/). Metabolite levels were regressed on exposure duration with starting age as an independent variable. The Purdue pegboard test scores were regressed on metabolite levels with age as an independent variable. All analyses were performed with a 95% confidence interval and p values <0.05 were considered to be statistically significant.

## Results

### Comparison of Mn exposure between smelters and control subjects

The mean value of airborne Mn levels in the smelters’ breathing zone was 0.40 mg/m^3^, which was higher than the maximum allowable concentration of Mn (0.2 mg/m^3^) according to the national standard set out by the Chinese Ministry of Public Health (TJ36-79). Airborne Mn levels for controls were below the detection limit.

The concentrations of Mn in erythrocyte and urine samples were significantly higher in smelters than in control subjects (p<0.001 and p = 0.002, respectively) (Table 1). No associations between erythrocyte or urine Mn levels and any of the Purdue pegboard tests, or with exposure duration, or with GABA levels were observed.

### Neurological exam

Despite the fact that all smelters were recruited from “healthy” workers (defined as never having sought evaluation or treatment for tremor, Parkinson disease or any motor dysfunction), the detailed neurological examination identified a subtle hand tremor in one of the smelters. Since this finding could be a possible effect of Mn exposure as described by literature, and we are specifically trying to explore the relationship between GABA levels and motor symptoms, this subject remained included in further data analyses. No other motor dysfunction was clinically diagnosed in any of the subjects.

### Changes in brain metabolites as a function of Mn exposure

**Figure 2:**
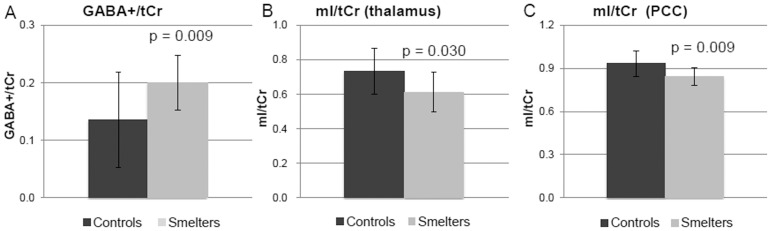
Histograms of GABA+/tCr levels in the thalamus and myo-inositol (the ratio of myo-inositol to total creatine (mI/tCr)) levels in the thalamus and posterior cingulated cortex (PCC). (A) Mean and standard deviation for GABA+/tCr in the thalamus of Mn-exposed smelters and controls (p = 0.009); (B) Mean and standard deviation for the ratio of myo-inositol to total creatine (mI/tCr) in the thalamus of Mn-exposed smelters and controls (p = 0.030); (C) Mean and standard deviation for mI/tCr in the PCC of Mn-exposed smelters and controls (p = 0.009).

Figure 1 depicts the VOIs for the two brain regions – thalamus (Figure 1A) and posterior cingulate cortex (PCC) (Figure 1B), as well as representative short-TE and GABA-edited spectra. Figure 1C shows a representative sagittal T1-weighted MRI brain image of a smelter, depicting the hyperintense signal associated with Mn deposition, especially in the globus pallidus. GABA+/tCr was significantly increased in the thalamus VOI of smelters compared to controls (p = 0.009) (Fig. 2A). This difference remained significant even if the one smelter with a subtle tremor was removed from the analysis (p = 0.037). Estimates for GABA+ concentrations from mean GABA+/tCr values were 1.2 mM for the smelters and 0.8 mM for the controls, assuming the concentration of tCr to be 6 mM [26]. The one smelter diagnosed with a subtle hand tremor had the highest GABA level (1.9 mM) of all subjects. Furthermore, the GABA+/tCr level displayed a significant association with exposure duration (R^2^ = 0.660, p = 0.039), indicating an Mn exposure-related increase in GABA levels. As displayed in Fig. 2B and 2C, myo-inositol (mI) levels in the thalamus and PCC, expressed as mI/tCr, were both significantly decreased (p = 0.030 and p = 0.009, respectively).

### Changes in motor deficits as a function of metabolite changes

**Figure 3:**
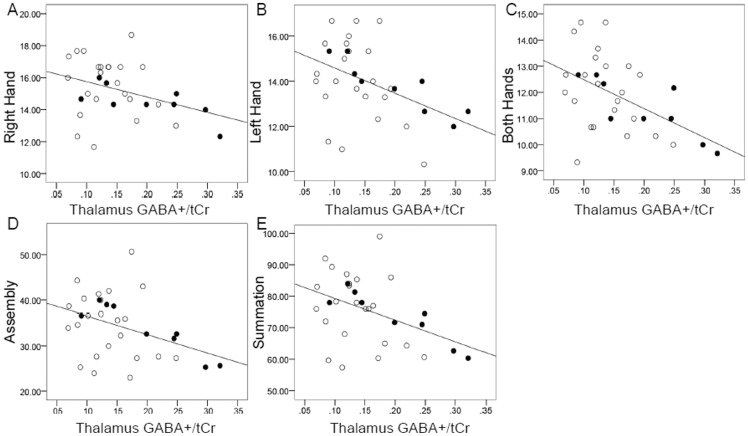
Multiple regression analyses (controlling for age) between GABA+/tCr in the thalamus and the (A) right hand (R^2^ = 0.259, p = 0.013), (B) left hand (R^2^ = 0.367, p = 0.001), (C) both hands (R^2^ = 0.428, p = 0.000), (D) assembly in 1 minute (R^2^ = 0.444, p = 0.000), and (E) summation of all Purdue pegboard test scores (R^2^ = 0.451, p = 0.000) for the combination of smelters (solid circles) and controls (open circles).

There were no significant group differences in the Purdue pegboard results between smelters and controls. In the smelters, strong inverse associations were found between an increase in GABA+/tCr and scores of *all* the Purdue pegboard tests (right hand R^2^ = 0.684, p = 0.013; left hand R^2^ = 0.843, p = 0.004; both hands R^2^ = 0.842, p = 0.004; assembly in 1 minute R^2^ = 0.891, p = 0.001; and summation of all test scores R^2^ = 0.902, p = 0.001). In the control group, weaker associations were observed between GABA+/tCr and pegboard scores, but all were significant except the right hand test (right hand R^2^ = 0.245, p = 0.060; left hand R^2^ = 0.306, p = 0.026; both hands R^2^ = 0.413, p = 0.005; assembly in 1 minute R^2^ = 0.443, p = 0.003; and summation of all test scores R^2^ = 0.444, p = 0.003). Significant associations were also found when the two groups were combined (right hand R^2^ = 0.259, p = 0.013, Figure 3A; left hand R^2^ = 0.367, p = 0.001, Figure 3B; both hands R^2^ = 0.428, p = 0.000, Figure 3C; assembly in 1 minute R^2^ = 0.444, p = 0.000, Figure 3D; and summation of all test scores R^2^ = 0.451, p = 0.000, Figure 3E). All these associations for the smelter group alone and the combined smelters and controls remained significant (p<0.05) if the one smelter with a subtle tremor was removed from the analysis. No other metabolite was associated with any of the pegboard results, nor were urine or erythrocyte Mn levels.

## Discussion

As noted above, this study confirmed our earlier finding that occupational Mn exposure among smelters results in increased GABA+/tCr levels in the thalamus. Moreover, *the current study revealed a significant association between fine motor performance and thalamic GABA levels*, suggesting that an altered thalamic GABA status may at least partially underlie the mechanism of motor deficits. The increased GABA levels in smelters thus might serve as biomarker for subtle motor changes in this occupationally Mn-exposed population.

The observation of Mn-induced GABA change has been controversial in the literature. Some rodent studies have found decreased GABA or no changes in GABA after Mn exposure [27]–[29], while others have observed an increased level of GABA [30]–[32]. For example, Bonilla (1978) first reported an Mn-induced increase in GABA in rat caudate nucleus. Lipe et al. (1999) subsequently found a significant increase of cerebellar GABA in a rat subchronic Mn exposure model wherein the rats received 20 mg/kg Mn per day for a month. An Mn exposure-induced increase in striatal GABA and an associated reduction of motor function were also observed in a pre-Parkinson’s rat model [32]. More recently, experiments on developing rats have provided evidence to support the hypothesis that Mn exposure may alter the GABA transporter and thereby inhibit the clearance of extracellular GABA, giving rise to an increase of GABA [33]. On the other hand, decreases in dopamine release have been measured in the striatum of the Mn-exposed non-human primates and in the caudate of Mn-exposed welders [34]–[36]. Such decreases can modify the GABAergic transmission of the direct and indirect basal ganglia pathways, involving globus pallidus, subthalamic nucleus and substantial nigra, to further influence thalamus GABA levels.

Mn accumulation may change the direct basal ganglia pathway by decreasing the GABA release from the striatum, causing disinhibition and thus excitation of the globus pallidus internal segment (GPi), or it may change the indirect pathway by increasing glutamate release from the subthalamic nucleus, possibly via altered GABA releases from striatal and pallidal neurons. While we cannot differentiate between effects on the direct versus indirect pathway, potentially increased GABA release from the GPi to the thalamus would cause increased inhibition of the thalamus. The motor nuclei of the thalamus project to the cortical motor area via an excitatory input [37]. The increase of GABA in the thalamus can inhibit the excitatory thalamic outflow to the cortical motor area, consequently disturbing the movement control mechanism. Thus, it seems likely that this change may underscore the observed association between increased thalamic GABA+ and decreased movement performance in the Purdue pegboard test.

In line with this hypothesis on the effects of increased inhibition of the thalamus, the current study found associations between thalamic GABA+ level and Purdue pegboard performances among all subjects (smelters and controls), suggesting that thalamic GABA+ may function as a marker for fine motor performance in general. Similar correlations were recently reported between sensorimotor cortex GABA concentration and nine-hole peg test scores in patients with multiple sclerosis [38]. The complex network of GABAergic modulation and other connections in the basal ganglia plays a key role in movement control. In our study the associations between increased thalamic GABA+ level and decreased fine motor performance were especially strong in the smelter group. The fact that the smelter who had the highest thalamus GABA+ level performed the worst in the Purdue pegboard test and was the same subject who was diagnosed with a subtle hand tremor further highlights the strong relationship between increasing GABA levels and motor deficits. Therefore, in smelters, thalamic GABA+ may work as a *biomarker for Mn-induced toxic effects*, i.e., a marker for subtle motor deficits.

The mean air Mn levels in this study seem to be comparable to the more highly exposed welders in Bowler et al. and Park et al [39], [40]; however, differences between exposure measurement techniques (area sampling versus personal sampling) need to be kept in mind. Unlike Bowler et al., Sen et al. and Wastensson et al. who all reported lower grooved pegboard test scores in welders [8], [41], [42], we did not find a significant difference in Purdue pegboard test scores between this smelter group and the control group, which is in agreement with Mergler et al. [43]. While scores of the two pegboard tests are known to reflect different aspects of motor function, coordination and cognitive speed with different sensitivity [44], there may be also an intrinsic difference in Mn exposure in a smelting versus a welding environment, which should be investigated in the future.

Myo-inositol is known to be synthesized mainly in glial cells in the brain [45] and thus is identified as a glial marker. We found a decrease of mI in both the thalamus and the PCC of smelters. Previous studies showed that Mn exposure can cause inflammation of glia [46]. As such, glial swelling or damage might therefore be the cause of decreased mI following Mn exposure, as suggested by Chang et al., who reported a similar decrease of mI in the anterior cingulate cortex in Mn-exposed welders [11].

The current study has a number of limitations. The sample size of the Mn-exposed smelters who underwent both the MRS and the Purdue pegboard test is small. Information on covariates, such as education of the subjects, their social and economic status, and their detailed medical history, among others, was not available for this study.

In conclusion, the results presented in this report confirm that occupational Mn exposure can lead to an elevated thalamic GABA level; a decrease in mI is a further indication of neurologic effects of Mn on selected brain regions. Moreover, it appears that GABA levels in the thalamus increase with increasing years of Mn exposure. The association between thalamic GABA and motor functioning supports the idea that MRS may become valuable for early diagnosis of Mn poisoning and that thalamic GABA may serve as a biomarker for subtle motor deficits in Mn-exposed populations.
